# Estimating Coextinction Risks from Epidemic Tree Death: Affiliate Lichen Communities among Diseased Host Tree Populations of *Fraxinus excelsior*

**DOI:** 10.1371/journal.pone.0045701

**Published:** 2012-09-25

**Authors:** Mari T. Jönsson, Göran Thor

**Affiliations:** Department of Ecology, Swedish University of Agricultural Sciences, Uppsala, Sweden; University of Kent, United Kingdom

## Abstract

At least 10% of the world’s tree species are threatened with extinction and pathogens are increasingly implicated in tree threats. Coextinction and threats to affiliates as a consequence of the loss or decline of their host trees is a poorly understood phenomenon. Ash dieback is an emerging infectious disease causing severe dieback of common ash *Fraxinus excelsior* throughout Europe. We utilized available empirical data on affiliate epiphytic lichen diversity (174 species and 17,800 observations) among 20 ash dieback infected host tree populations of *F. excelsior* on the island Gotland in the Baltic Sea, Sweden. From this, we used structured scenario projections scaled with empirical data of ash dieback disease to generate probabilistic models for estimating local and regional lichen coextinction risks. Average coextinction probabilities (*Ā*) were 0.38 (95% CI ±0.09) for lichens occurring on *F. excelsior* and 0.14 (95% CI ±0.03) when considering lichen persistence on all tree species. *Ā* was strongly linked to local disease incidence levels and generally increasing with lichen host specificity to *F. excelsior* and decreasing population size. Coextinctions reduced affiliate community viability, with significant local reductions in species richness and shifts in lichen species composition. Affiliates were projected to become locally extirpated before their hosts, illuminating the need to also consider host tree declines. Traditionally managed open wooded meadows had the highest incidence of ash dieback disease and significantly higher proportions of affiliate species projected to go extinct, compared with unmanaged closed forests and semi-open grazed sites. Most cothreatened species were not previously red-listed, which suggest that tree epidemics cause many unforeseen threats to species. Our analysis shows that epidemic tree deaths represent an insidious, mostly overlooked, threat to sessile affiliate communities in forested environments. Current conservation and management strategies must account for secondary extinctions associated with epidemic tree death.

## Introduction

**Figure 1:**
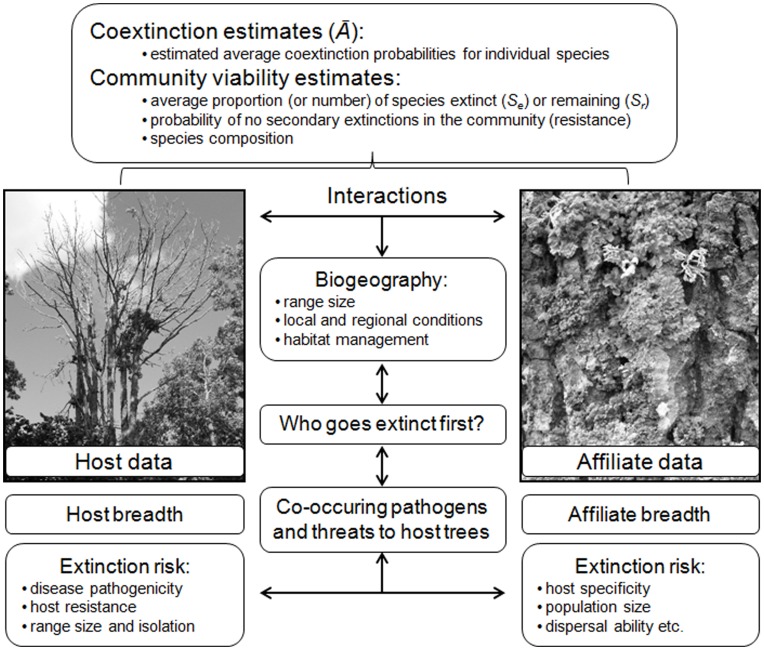
A conceptual framework for the components influencing affiliate coextinction and community viability during tree epidemics, adapted after Moir *et al.* [20]. Coextinction and community viability is primarily influenced by host trees, affiliate species, and their interactions. These variables are in turn influenced by several factors (see text). The left photo show an ash dieback diseased tree (classified as dying) surrounded by healthy *F. excelsior* on Gotland Island in 2009.

Pathogens are individually, or in association with other factors, increasingly implicated in the decline, threats and extinction of a wide range of species and the degradation of ecological systems throughout the world [1]–[7]. Regionally throughout Europe, the fungal pathogen *Hymenoscyphus pseudoalbidus* (anamorph *Chalara fraxinea*) [8] has been causing severe dieback of common ash *Fraxinus excelsior* in wooded stands of all ages and [9]–[12]. Such emerging infectious diseases (EIDs) [1] threaten not only their immediate host but also have serious, often unknown, cascade effects on species composition, structure and function of terrestrial ecosystems [6], [13], [14]. Yet, studies of secondary extinctions and community-level changes caused by epidemic tree death are almost nonexistent [14]. This is due to limited quantitative baseline data on pre-epidemic conditions [3], [6] and the lack of rapid responses in terms of targeted funding programs and anticipatory scenario planning [15]. General strategies (i.e., scenario planning and exploratory risk analysis) for discerning the circumstances under which EIDs of trees cause secondary extinctions, threats and community-level changes are urgently needed [3]. Such predictive knowledge is necessary for making realistic estimates of extinction risks on which to base remedial conservation and management options. Community viability analysis [16], [17] may provide some of these tools, but its utility awaits empirical evaluation. Probabilistic models suggest that “coextinction” may be the most common form of global biodiversity loss [18]. The terms coextinction and cothreatened refer to the phenomena when the loss or decline of a host species results in the loss or endangerment of other species that depend on it, potentially leading to cascading effects across trophic levels [18]–[20]. This coextinction threat is amplified with increasing host specificity of the affiliate species [18]. Paradoxically, coextinction remains a poorly quantified phenomenon with few historical or current coextinction events actually recorded [18]–[20]. Coextinction models using empirical data based on a variety of localities and host specificity distributions, as well as interactions and synergies from other drivers of species loss (e.g., land-use change, climate change, pathogens, and invasive species) would give more accurate extinction estimates [19], [20]. In this study we utilized available empirical data on affiliate epiphytic lichen diversity among 20 ash dieback infected host tree populations of *F. excelsior*. From this, we generated probabilistic models for coextinction and threats to local and regional affiliate lichens resulting from two outcome scenarios of ash dieback disease. Secondary effects on community viability (*sensu* [16], [17]) were then analyzed at both local and regional perspectives (Fig. 1 *sensu* 20). We furthermore explored coextinction probabilities in relation to: (i) lichen traits such as tree host specificity to *F. excelsior* and vulnerability (e.g., number of occupied stands, dispersal mode, and red-list status according to Gärdenfors [21]), and (ii) three management categories; unmanaged closed forest; grazed semi-open forests; and open traditionally managed wooded meadows with pollarding of ash trees, mowing and hay gathering.

## Methods

### Ethics Statement

All necessary permits were obtained for the described field studies.

### Study Area and Selection of Study Sites

Study stands were located on the island Gotland in the Baltic Sea about 90 km east of the Swedish mainland. Gotland is the largest island in the Baltic Sea (c. 3,151 km^2^ with 57,000 inhabitants in 2007). The island is located in the transitional hemiboreal vegetation zone, whereof both coniferous forests and deciduous woodlands thrive. Mean annual precipitation is 500–600 mm and mean monthly temperatures range from –1°C in February to 16–17°C in July (Swedish Meteorological and Hydrological Institute, records 1961–2009).

The study sites were 20 wooded stands previously inventoried for epiphytic lichens in 1989–1991. These were evenly distributed throughout the island, about 1–9 ha in size, and included at least 50 *F. excelsior* trees. Seven were managed as traditional wooded meadows (i.e., mowing, hay gathering, pollarding of *F. excelsior*) with low average canopy closure and tree densities, six were managed by grazing and with semi-open canopies, and seven were unmanaged closed canopy forests (Table 1). Lichen assemblages therefore represent a variety of wooded stand conditions with *F. excelsior*. The dominant trees in the study sites were *F. excelsior* and pedunculate oak *Quercus robur*, with small-leaved elm *Ulmus minor*, birch *Betula* spp. and Scots pine *Pinus sylvestris* as subordinate trees.

**Table 1:** Lichen community values and stand characteristics of the 20 wooded study sites on Gotland, Sweden.

		All lichens		Red-listed		Stand characteristics (%)						Post-epidemic optimistic scenario						Post-epidemic likely scenario					
Management type	No. of trees	S.R.	No. of Ind.	S.R.	No. of Ind.	Size (ha)	Canopy closure	Poll. ash	Ash	Oak	Other	% of trees	*R*	Res^1^	*S_e_* ^2^	Res^3^	*S_e_* ^4^	% of trees	*R*	Res^1^	*S_e_* ^2^	Res^3^	*S_e_* ^4^
***Unmanaged***																							
Binge	22	65	407	1	2	2.0	60	0	48	38	14	0	NA	1.0	0.0	1.0	0.0	33	0.10	0.0	9.5	0.0	3.0
Aumunds	15	86	337	8	16	5.8	75	0	37	51	12	0	NA	1.0	0.0	1.0	0.0	50	0.26	0.0	20.0	0.0	10.0
Ellstädaränget	17	48	236	5	10	6.0	95	0	32	36	32	23	0.02	0.0	7.3	0.2	4.0	77	0.63**	0.0	39.0	0.0	9.0
Vallbys	15	53	217	5	14	3.1	60	0	50	50	0	25	0.02	0.0	8.7	0.3	4.0	68	0.49*	0.0	35.3	0.0	13.0
Kullingbos	21	74	358	6	6	4.5	60	0	44	40	6	28	0.09	0.0	11.1	0.0	7.0	68	0.50**	0.0	33.1	0.0	20.0
Elinghem	16	58	231	1	1	4.9	80	0	30	38	32	36	0.10	0.0	14.9	0.1	3.0	71	0.50*	0.0	37.9	0.0	7.0
Kulbjershagen	20	53	265	4	7		75	0	43	49	8	37	0.12	0.0	10.6	0.2	4.0	71	0.53**	0.0	30.0	0.0	9.0
Average	18	62	293	4	8	4.4	72	0	41	43	15	21	0.07	0.3	7.5	0.4	3.1	63	0.43	0.0	29.3	0.0	10.1
***Grazed***																							
Skäggs	18	74	443	4	8	4.5	60	17	38	38	24	0	NA	1.0	0.0	1.0	0.0	17	0.01	0.0	4.6	0.2	1.0
Bjärs	21	73	483	5	7	1.8	65	0	40	35	20	24	0.03	0.0	5.6	0.1	4.0	58	0.36*	0.0	20.0	0.0	14.0
Bäckstäde	23	96	718	7	25	5.5	55	0	48	40	12	32	0.12	0.0	7.3	0.0	4.0	76	0.61**	0.0	30.2	0.0	16.0
Haltarve	24	80	532	2	6	3.0	60	66	50	35	15	48	0.26	0.0	17.0	0.0	9.0	78	0.65**	0.0	35.9	0.0	16.0
Oggesänge	19	57	278	4	22	7.5	75	26	41	48	11	49	0.26	0.0	18.0	0.0	7.0	73	0.56**	0.0	34.5	0.0	11.0
Tomsarve	21	81	497	4	7	1.0	60	0	46	48	6	60	0.37*	0.0	22.0	0.0	12.0	93	0.85**	0.0	57.9	0.0	25.0
Average	21	77	492	4	13	3.9	63	18	44	41	15	36	0.21	0.2	11.7	0.2	6.0	66	0.51	0.0	30.5	0.0	13.8
***Traditionally***																							
Kue	20	77	457	3	4	0.8	55	21	43	17	40	13	0.02	0.2	2.9	0.4	1.0	48	0.26	0.0	15.1	0.0	8.0
Lojsta	24	73	392	8	23	2.9	50	64	53	36	11	40	0.17	0.0	12.2	0.0	5.0	82	0.70**	0.0	42.4	0.0	19.0
Laxare	21	53	291	6	19	5.3	50	60	39	33	28	44	0.18	0.0	14.1	0.0	6.0	87	0.78**	0.0	54.2	0.0	15.0
Mästerby	24	96	449	9	23	6.3	50	11	45	36	19	45	0.21	0.0	17.5	0.0	15.0	94	0.85**	0.0	64.2	0.0	25.0
Vall	18	86	452	2	4	7	50	25	47	45	8	51	0.27	0.0	17.2	0.0	6.0	89	0.82**	0.0	54.6	0.0	14.0
Fide Annex	17	72	303	9	19	2.3	55	85	38	49	13	56	0.36*	0.0	24.8	0.0	10.0	92	0.86**	0.0	69.4	0.0	24.0
Hulte Kruppar	20	71	314	4	11	4	45	82	42	42	16	61	0.40*	0.0	25.6	0.0	11.0	98	0.92**	0.0	75.5	0.0	21.0
Average	21	75	380	6	15	4.1	51	50	44	37	19	44	0.23	0.0	16.3	0.1	7.7	84	0.74	0.0	53.6	0.0	18.0

Site-level pre-epidemic lichen community values from inventories in 1989–1991; showing the number of *Fraxinus excelsior* trees inventoried for lichens, species richness (S.R.) and abundance values (No. of Ind.) of all lichens and red-listed species, respectively. Stand characteristics for each site and management category; showing stand size (ha), percentage values of canopy closure, fraction of *F. excelsior* population with signs of recent pollarding (pollarded within the last two decades), and percentage tree species composition. Projected post-epidemic site-level lichen community values; showing the percentage of the *F. excelsior* population classified as dead or dying (optimistic scenario) and infected (likely scenario), average ANOSIM R values comparing pre-epidemic and projected post-epidemic local lichen species composition on *F. excelsior* tree populations, the projected probability values of no local coextinctions (Res) and the average percentage of extinct species (*S_e_* ) among *F. excelsior* tree populations and all tree species in the respective post-epidemic community.

* bonferroni-corrected *p*<0.05, ** b.f. *p*<0.001 ^1^the probability of no coextinctions and ^2^the average proportion (%) of extinct species (*S_e_*) on ash *F. excelsior* in the post-epidemic community ^3^the probability of no coextinctions and ^4^the average proportion (%) of extinct species (*S_e_*) when including occurrences on all tree species.

### Baseline Data on Trees, Pathogens and Selection of Outbreak Scenarios

Disease incidence data of ash dieback and *F. excelsior* was collected from the 20 study sites in July 2009, when wilting symptoms are clearly visible in the field. A tree was defined as a living lignified vascular plant with a circumference of 30 cm or more at 1.5 m above the ground. For each tree we recorded the circumference at 1.5 m height and the geographical position (XY coordinates) to allow reiteration. In small open stands with good visibility all *F. excelsior* trees were inventoried, but in closed-canopy unmanaged stands *F. excelsior* was inventoried in randomly placed circular sampling plots with a radius of 20 m. Ash dieback disease was present in all stands inventoried. In each study site a minimum of 50 *F. excelsior* trees were inventoried for disease symptoms and classified as (1) dead, with no living foliage, and cankers on bark and branches that had started to crack and fall off; (2) dying, with stem necroses and cankers, advanced death of branches, almost complete crown dieback with only a few branches of clumped foliage on shortened internodes (Fig. 1); (3) infected, with substantial top-dry or crown dieback, premature defoliation and wilted or dead foliage; or (4) visually healthy trees with no apparent disease symptoms. A total of 1,066 *F. excelsior* trees were inventoried for ash dieback disease.

Two ash dieback outbreak scenarios (structured accounts of possible futures) that differed in the degree of tree mortality were selected based on 2009 disease symptoms: (1) a *most likely scenario* with tree mortality permutations based on proportions of dead, dying and infected trees, and (2) a *most optimistic scenario* based on proportions of dead and dying trees within sites. The majority of the infected trees are likely to succumb to the disease within approximately 10 years [8]–[12], [22].

### Baseline Data on Epiphytic Lichen Communities

Baseline data on total epiphytic lichen diversity was inventoried in 1989–1991, before the occurrence of ash dieback in 2003. Transects were established in each wooded stand following it along the longest possible line. Each transect was then divided in length by 30 and at each of the 30 subdivisions the nearest tree was inventoried. Tree species, circumference at 1.5 m above the ground, and all lichens (crustose, foliose, fruticose) on the trunk and the branches up to two metres height were recorded. Only presence-absence data of the lichen species was registered. Furthermore, at every other sampling point the nearest *F. excelsior* and *Q. robur* tree was examined. Thus, a minimum of 15 *F. excelsior* and 15 *Q. robur* were investigated in each site (the two dominant tree species), together with a random sample of 30 trees of all tree species. For further details on lichen inventories we refer to Thor *et al.* [23]. More than 7,600 lichen observations, representing 174 taxa were recorded on 386 *F. excelsior* trees. In addition, over 10,200 observations of the same lichen species were made on 374 *Q. robur* and 164 trees of other tree species. The nomenclature for lichens follows Santesson *et al.* [24] and vascular plants Krok and Almquist [25].

### Estimation of Affiliate Coextinction and Community Viability

In our affiliate coextinction models, we considered empirical matrices of host tree species and their affiliate lichens, and examined the consequences for affiliate diversity of removing, at random, a given number of host trees according to its site specific disease incidence fraction. This approach makes the assumptions that individual host trees go extinct randomly within sites and that the sampling of tree and lichen relationships was appropriate. Given the speed and course of which the disease spreads, we assumed that affiliates do not adapt or “switch host” as *F. excelsior* become rare [19]. We used a random permutation procedure to project tree mortality perturbations for each scenario and locality. We projected 100 lichen assemblages for each locality and scenario, resulting in 4,000 projected assemblages (i.e., the post-epidemic communities) to be compared with the original epiphytic lichen assemblages prior to the appearance of ash dieback (i.e., the pre-epidemic communities). We repeated the mortality permutations for *F. excelsior* populations individually, as well as for all tree species populations (including trees such as *Q. robur*) to assess the relative significance of *F. excelsior* mortality in mixed wooded stands with multiple host trees.

We used community viability analysis [16], [17] to quantify (i) the average proportion of species extinct (*S_e_*) or remaining (*S_r_*) in the post-epidemic community; (ii) the probability that the proportion of species remaining in the community falls below a particular level following a certain tree-death scenario (quasi-collapse risk *sensu* Ebenman *et al.* [16] and (iii) the probability that there will be no extinctions in the community following a tree-death scenario (i.e., resistance). We produced risk curves according to Ebenman *et al.* [16], [17] for local lichen communities on *F. excelsior* to visualize the risk and extent of extinctions following the loss of host trees. Generating a risk curve starts with calculating the frequency of community replicates with 0 to *S_o_* − 1 number of species remaining (*S_r_*), where *S_o_* is the number of species in the original community. From this, the cumulative number of replicates with < *S_o_* − 1, *S_o_* − 2,…, species remaining is obtained and rescaled (dividing by the total number of replicates), to get the probability that loss of the host will result in a post-epidemic community with less than *S* species. This is the quasi-collapse risk *p*(*S_r_* < *S*). The graph of quasi-collapse risks is the risk curve, and the steeper this curve is, the greater the loss of species is.

Local coextinction probabilities for the 174 study species were calculated from the 100 *F. excelsior* mortality permutations of each study site, considering both *F. excelsior* tree populations and mixed tree species populations. Average affiliate coextinction probabilities (*Ā*) of individual species (see Appendix S1 in supporting information) were calculated from scenario projections based on all study sites where the individual species was present. A cothreatened affiliate was defined according to its projected average extinction probability and the International Union for Conservation of Nature (IUCN) criteria threshold value designed to estimate extinction risk [21]. We applied a threshold value according to that of *Endangered* (EN) category species, where a quantitative analysis indicate that the probability of extinction in the wild (in this case our study sites on Gotland) is at least 20% within 20 years. After reviewing the relevance of the projected list of cothreatened species, one species was subsequently omitted due to known high occurrence on alternative trees and another two species were omitted due to taxonomic revisions (see Appendix S1).

Attributes of specialization (host specificity to *F. excelsior*), rarity (small population size and distribution range), and dispersal mode (sexual dispersal via spores or asexual dispersal via fragmentation, isidia, conidia etc.) may also increase vulnerability to coextinction among lichens in the affiliate community. Hence, we tested whether *Ā*, generated under the optimistic and likely scenarios for all tree species and lichens, were explained by lichen host specificity to *F. excelsior*, number of occupied stands (1–20), dispersal mode (sexual or asexual dispersal according to Smith *et al*. [26]), and Red-List status (red-listed or not red-listed according to Gärdenfors [21]). Host specificity of individual lichens, or degree of polyphagy, was measured as the fraction of lichen records occurring on *F. excelsior* in relation to the total number of observations on all host trees (based on data from the random tree inventories). In the subsequent analysis, the response variable *Ā* is a proportion measure that take on values bound between 0 and 1. The beta distribution is more appropriate for modeling such data since it adequately describes the frequency distribution of proportions and does not require transformation of the response variable [27]. Hence, the statistical analysis of variables influencing *Ā* was conducted in the statistical program R version 2.14.2 by beta regression using the package Betareg [28]. Numeric explanatory variables (host specificity and number of occupied stands) were standardized by dividing by two standard deviations and binary variables (dispersal mode and Red-List status) were coded as 0/1 [29]. The pseudo-R^2^ value, which is the squared correlation of the linear predictor and link-transformed response, was calculated and compared [27]. Model building was started by testing each explanatory variable separately, which showed that all variables were significant on an individual basis. We then used the number of occupied stands as the starting variable in the models, and the remaining significant variables were then added one by one, in order of explained deviance. We also used beta regression to test site-level proportions of extinct lichen species (*S_e_*) on trees (all species included) in relation to site management category.

### Estimation of Species Composition

We used one-way analyses of similarity ANOSIM [30], [31] in the PAST software package version 1.57 [32] to investigate differences in lichen species composition of pre-epidemic communities and post-epidemic communities on *F. excelsior*. In each case, the analyses were based on a Bray–Curtis similarity matrix built on the tree-level presence–absence of each species in each locality [31]. Each projected post-epidemic community for a specific locality and scenario were contrasted with its pre-epidemic community. ANOSIM generates an *R*-statistic which gives a measure of how similar groups are: values most commonly range from 0–1. A large positive *R* close to one signifies large differences between groups, while a value close to zero indicates there is little difference between groups [32]. Levels of significance *p* of the differences between assemblages were obtained by a permutation procedure (with 10, 000 replicates) on the similarity matrices [32].

Nonmetric multidimensional scaling (NMDS) in the program PAST [32] was used to generate a visual configuration of the significant compositional differences on *F. excelsior* at the most likely scenario (see Fig. S1). The NMDS plot was generated from a Bray–Curtis similarity matrix of untransformed data of average presence-absence probability values for each locality based on projected data (n = 100), where average presence probabilities ≥0.5 were treated as presences and <0.5 as absences. The algorithm implemented in PAST attempts to place the data points in a two- or three-dimensional coordinate system such that the ranked differences are preserved [33]. The program may converge on a different solution in each run, depending on the random initial condition. Each run is a sequence of 11 trials, from which the one with smallest stress (mismatch between the rank order of distances in the data and the rank order of distances in the ordination) is chosen. Stress levels below or close to 0.10 are considered as good representatives of the data with little danger of drawing false inferences [34]. The solution is automatically rotated to the major axes (2D and 3D).

## Results

From 1,066 *F. excelsior* trees, 28% were healthy, 36% infected, 18% dying and 16% dead. The percentage of infected trees varied between study sites with an average of 71% (95% CI ±9.9) of trees infected with the disease across sites (Table 1). Hence, the most likely scenario used average mortality rates of 71%, given that infected trees rarely recover, and the most optimistic scenario used average mortality rates of 34%, based only on dead and dying *F. excelsior*. Notably, low disease incidences were recorded in two localities with only 17% and 33% of *F. excelsior* infected. On average 84% of *F. excelsior* trees displayed symptoms of infection in traditionally managed sites, compared to 63% in unmanaged sites and 66% in grazed sites (Table 1).

**Figure 2:**
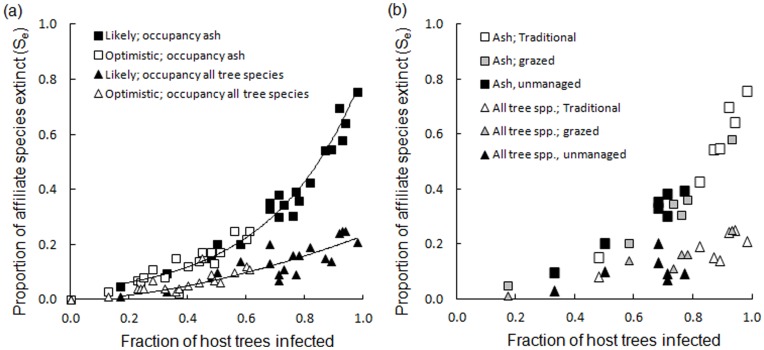
Average proportion of affiliate lichen species projected to go extinct (*S_e_*). (a) *S_e_* as a function of the fraction of host trees infected at each study site, given mortality permutations of 2009 levels of dead and dying *F. excelsior* (unfilled; optimistic scenario) and all infected *F. excelsior* (filled; likely scenario). Squares represent *S_e_* among lichen communities on ash *F. excelsior* and triangles represent *S_e_* among lichen communities on all tree species. (b) Average proportion of affiliate lichen species projected to go extinct (*S_e_*) in each management category under the most likely scenario.

We projected significant average reductions in the number of surviving affiliate lichens on *F. excelsior* tree populations at both the most optimistic (average site-level species loss 12% and 95% CI ±3.5) and the most likely scenario (average loss 38% and 95% CI ±9.4), compared with pre-epidemic communities (two-tailed paired *t*-tests; *t*-values >3.8, *p*-values ≤0.001, df = 19). The average proportion of affiliate lichen species projected to go extinct (*S_e_*) was relatively low at the most optimistic scenario but increased greatly at the most likely scenario with an increasing fraction of the *F. excelsior* population lost (Fig. 2a). Extinction curves were linear for the optimistic scenario (not shown in graphs for ease of interpretation), exponential at the most likely scenario for *F. excelsior* (y = 0.033e^3^.^201x^, R^2^ = 0.97), and a power function at the most likely scenario for all tree species (y = 0.230x^1^.^701^, R^2^ = 0.85). Hence, as the number of host tree infections and declines increased, the number of extinctions increased at an accelerating rate when approximately 60–65% of the local host population of *F. excelsior* was lost (Fig. 2a). This curvilinear relationship between host and affiliate extinction levels was most pronounced for *F. excelsior* populations; as expected, affiliated species with low host specificity to *F. excelsior* were more resistant to epidemic tree death when considering their survival on alternative host trees in the mixed wooded stands. Proportions of affiliate lichen species projected to go extinct (*S_e_*) were greatest in the traditionally managed stands (Fig. 2b, Table 1, 2b).

**Table 2:** Beta regression model results of average coextinction probabilities (*Ā)*.

Response variable	Explanatory variable	Estimate	SE	*z*-values	*p*
***Average coextinction probabilities (Ā)***	Intercept	−3.161	0.269	−11.741	<0.000
Optimistic scenario (Pseudo R^2^ = 0.36)	Number of occupied sites	−0.509	0.174	−2.932	0.003
	Host specificity	0.931	0.178	5.224	<0.000
	Dispersal mode (asexual as ref.)	0.417	0.163	2.553	0.011
	Red-listed (not red-listed as ref.)	−0.210	0.239	−0.880	0.379
***Average coextinction probabilities (Ā)***	Intercept	−2.819	0.264	−10.693	<0.000
Likely scenario (Pseudo R^2^ = 0.49)	Number of occupied sites	−0.781	0.175	−4.471	<0.000
	Host specificity	1.917	0.187	10.274	<0.000
	Dispersal mode (asexual as ref.)	0.168	0.163	1.029	0.304
	Red-listed (not red-listed as ref.)	−0.216	0.238	−0.906	0.365
***Average proportions of extinct lichens (S_e_)***	Intercept	−3.509	0.415	−8.449	<0.000
Optimistic scenario (Pseudo R^2^ = 0.14)	Grazed (unmanaged as ref.)	0.516	0.501	1.029	0.304
	Traditional (unmanaged as ref.)	1.153	0.467	2.471	0.014
***Average proportions of extinct lichens (S_e_)***	Intercept	−2.117	0.239	−8.864	<0.000
Likely scenario (Pseudo R^2^ = 0.15)	Grazed (unmanaged as ref.)	0.132	0.335	0.394	0.694
	Traditional (unmanaged as ref.)	0.639	0.304	2.104	0.035

Beta regression models explaining average coextinction probabilities (*Ā)* of 174 lichen species occurring on trees (all tree species included) in 20 wooded stands affected by ash dieback disease, under the most optimistic scenario and the most likely scenario of *F. excelsior* tree death. Beta regression explaining average site-level proportions of extinct lichen species (*S_e_*) on all trees in relation to site management category.

**Figure 3:**
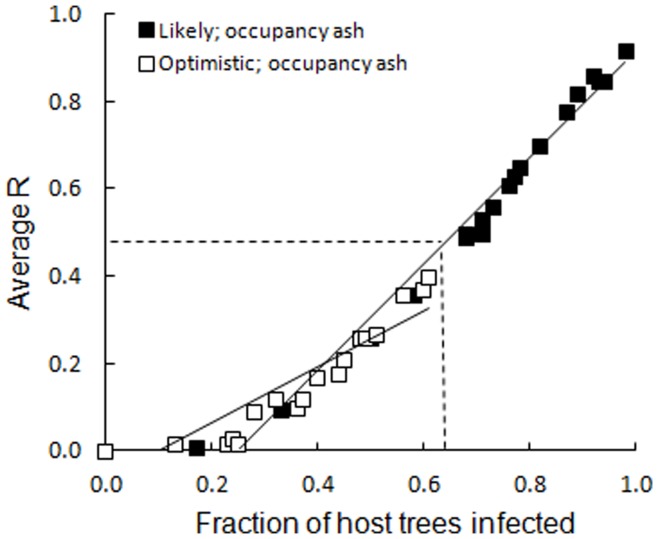
Lichen species composition among ash dieback infected host tree populations of *F. excelsior*. Average ANOSIM R values for comparison between 20 unaffected pre-epidemic local lichen species composition on *F. excelsior* tree populations and projected assemblages subjected to optimistic and likely tree mortality perturbations. Data represent average R values of 100 projections for each study site. R-values around 0.5 (above the dashed line) indicate clear differences in species composition between groups.

Average R-values from the ANOSIM (Fig. 3) and NMDS ordinations (see Fig. S1) pointed to significant shifts in species composition at the most likely scenario for *F. excelsior* populations. In addition to reductions in species richness, significant changes in local lichen species compositions occurred when approximately 60–65% of the local *F. excelsior* populations were lost. The most optimistic scenario projections of lichen communities on *F. excelsior* generally maintained resemblance with pre-epidemic communities (average R <0.5, *p*-values >0.05) (Fig. 3).

**Figure 4:**
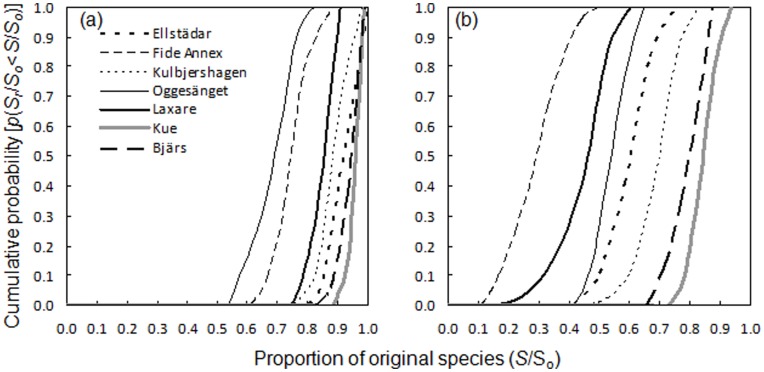
Risk curves for seven local lichen communities on *F. excelsior* subjected to ash dieback. The curves show the cumulative probability that the proportion of species remaining in the community falls below a certain proportion of the original species following the most optimistic (a) and the most likely scenario (b) of ash dieback mortality. Each curve is computed from 100 replicate communities. Remaining communities fall within the current range, but are not shown to ease visual interpretation.

At site-level, we produced risk curves for lichen communities on *F. excelsior* to visualize the risk of local extinctions following the loss of host trees. These risk curves (for a subset see Fig. 4) illustrate the substantial, although variable, extent of local extinctions at the most likely scenario. The probability that there will be no extinctions (resistance) following even the most optimistic scenario was virtually zero for the vast majority of sites, even when considering lichen occurrences on multiple host trees (see Table 1).

**Figure 5:**
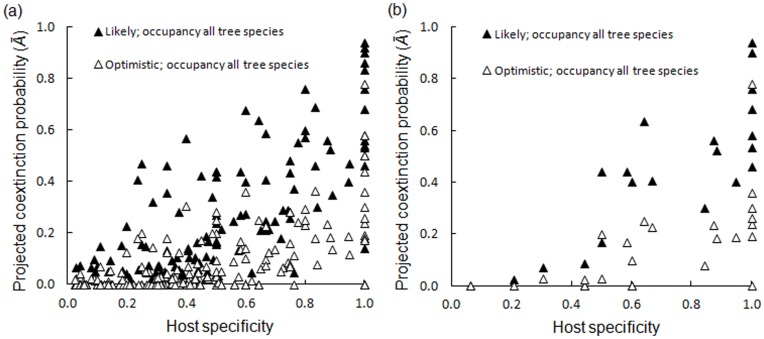
Projected average coextinction probabilities (*Ā*) as a function of host specificity. (a) *Ā* for all 174 affiliate lichen species at the most optimistic and the most likely scenarios of ash dieback disease, and (b) for the 23 lichen species currently red-listed in Sweden [21]. Beta regression model results for these relationships are shown in Table 2.

Estimated average coextinction probabilities (*Ā)* were 0.38 (95% CI ±0.09) for lichens occurring on *F. excelsior* and 0.14 (95% CI ±0.03) when considering lichen persistence on co-occurring tree species (Table 1). *Ā* increased with the host specificity of the affiliate (Fig. 5a) and a decreasing number of occupied sites (Table 2a). Red-listed species with large proportions of their population on *F. excelsior* were at greater risk of extinction than red-listed species co-occurring on multiple host trees (Fig. 5b). Considering occurrences on all tree species and the most likely scenario, *Ā* was on average 0.40 for red-listed species, compared with an average coextinction probability of 0.16 for the remaining Least Concern category species. When analyzed individually, red-listing did have a significant effect on increased *Ā* (both scenarios; beta regression *z*-values >2.7 and *p*-values >0.01). However, this effect was not significant when analyzed together with the other explanatory variables in the beta regression analysis (Table 2a). A total of 100 sexually dispersed species (via spores; average *Ā* for all trees was 0.10 at optimistic scenario and 0.24 at likely scenario) did have weakly significantly higher *Ā* compared with a total of 74 asexually dispersed species (average *Ā* of 0.05 at optimistic scenario and 0.10 at likely scenario) at optimistic scenario, but no such significant effect at the likely scenario (Table 2a). Around 35% of the affiliate lichens were cothreatened in the most likely scenario, considering occurrences on all tree species and the IUCN Red-List criteria for Endangered species. Only 27% of these cothreatened species were already nationally red-listed (Appendix S1).

## Discussion

The incidence of dead, dying and infected *F. excelsior* varied between stands, however, at proportions similar to that previously described in the literature (e.g., [9], [10]). Molecular research and pathogenicity tests also show that an average infection of approximately 70% fall within the range of variation in disease susceptibility and mortality proportions of *F. excelsior* trees [11], [12], [22], [35]. Only a small fraction of the *F. excelsior* population is likely to survive due to inheritable resistance mechanisms [35] or beneficial phenological traits such as early leaf senescence [22]. Based on these disease assumptions, our scenario projections clearly showed that coextinctions from ash dieback disease represent an insidious threat to affiliate lichen community viability in forested environments.

Our results pointed to a curvilinear relationship between proportions of affiliate species lost and fraction of host trees lost at the most likely scenario of ash dieback disease. Local extinctions occurred at an accelerated rate when a certain fraction of host trees was lost. Similar curvilinear relationships have been reported for affiliate species with multiple hosts such as butterflies and their larval host plants [18], [19]. This curvilinear relationship may also explain, at least partly, why so few coextinctions and cothreatened species have been documented [18], [19]. Our results clearly show that tree affiliates can go locally extinct before their hosts, which illuminate the need for coextinction models to incorporate not only host extinctions but also declines in host populations (Fig. 1) [20]. In addition to reduced local species richness, we showed that accelerated extinctions imposed by severe epidemic tree death (>60%) also lead to significant changes in lichen species composition on *F. excelsior*. These results high-lights the importance of evaluating a breadth of affiliate species and community-level changes, as well as extinctions of individual species (Fig. 1). Community assembly is important, given that species with different traits often takes on diverse functional roles in a community (e.g., [36]). Important functions by lichens, such as photosynthesis and nutrient cycling, can be discontinued. Insects and mollusks dependent on certain lichens for shelter and food resources may be adversely affected [37], [38]. Such wider community impacts can in turn cause a cascade of species declines, extinctions or other disruptions. Species functional redundancy in epiphytic post-epidemic communities has never been studied.

Estimated average coextinction probabilities (*Ā*) were dependent on local management factors and *F. excelsior* disease resistance levels, but also affiliate species traits. Species with narrow niches (few alternative host trees) and small population size (few occupied sites) were more likely to become coextinct. This is not surprising considering that many lichens have small population sizes and specific habitat requirements in relation to tree identity, age and size (e.g., [23]). Also, many epiphytic organisms such as lichens and bryophytes, as well as many wood and bark-inhabiting fungi and insects, are characterized by a patch-tracking metapopulation structure which has connectivity-dependent colonizations and local extinctions caused by the turnover of the tree, i.e., “patch” [39]. The long-term survival of these species is dependent on the continuous presence of long-lived broadleaved deciduous trees in the near vicinity (*sensu* [40]). Dispersedly restricted affiliates with patch-tracking metapopulation structure and few host trees are clearly more vulnerable to coextinction when faced by dramatic local host-tree reductions [41]. A tendency for higher *Ā* among sexually dispersed species was probably related to an average higher host specificity to *F. excelsior* among these species (*s* = 0.51 at the likely scenario) compared with asexually dispersed species (*s* = 0.35). Hence, sexually dispersed species likely have narrower realized host tree niches since their fungal mycobionts (spores) need to re-lichenize with a suitable algal and/or cyanobacterial photobiont partner for successful establishment of symbiotic phenotypes. Vegetative dispersal of symbiotic partners by joint algal and fungal propagules, on the other hand, can be considered a more efficient strategy for rapid colonization of available habitats and to circumvent low symbiont availability on suitable host trees.

Epidemic tree death impose temporal bottlenecks with low densities of old host trees which may last several hundreds of years, given that resistant tree populations might rebound at sufficient densities and spatial patterns. In view of the foregoing, it is imperative that localities with infection levels below approximately 60% become identified as particularly valuable areas for remedial conservation and management. These localities maintained comparatively viable affiliate communities in terms of species composition and local coextinction risk curves, hosting more intact species pools for future recovery. Natural or artificial selection and replanting to favor the remaining healthy host trees at these sites, are important measures for future maintenance of *F. excelsior* and affiliate biodiversity. The average high proportion (50%) of recently pollarded *F. excelsior* and low canopy closure (51%; as indicative of lower host tree densities) in traditionally managed stands, compared with unmanaged (72% canopy closure and no pollarding) and grazed sites (63% canopy closure and 18% pollarding), may explain the higher incidence of ash dieback disease and subsequent higher extinction risks in traditionally managed stands (Table 1 and 2b). Pollarding removes the upper branches of the tree, promoting a dense head of foliage and branches. The intense sprouting of new foliage may be more susceptible to infections from *H. pseudoalbidus*, although this remains to be further studied. Traditionally, trees were pollarded for fodder to feed livestock or for wood, but today this is done mainly as a conservation measure to promote important trees structures like cavities and slow tree growth. Hence, aiming to pro-long the life of *F. excelsior* through conservation pollarding (often funded through EU conservation management policies) may only be counterproductive when faced with ash dieback disease. This represent a major conservation and management challenge since traditionally managed stands generally also host more red-listed species than unmanaged and grazed sites (Table 1). The low pseudo-R^2^ values of the beta regression models for *S_e_* (ranging from 0.14 to 0.15), however, suggest a lot of unexplained variation due to factors other than management category.

There is no standard model for estimating the potential loss of species through coextinction. The simplest approach assumes unique host dependency (i.e., monophagy) and a linear 1∶1 ratio relationship between coextinctions of affiliates and their hosts. Here we incorporated host specificity (*s*) in our models as a fractional measure.We considered this to be an appropriate measure for estimating local and regional coextinction risks, but acknowledge that quantifications of host specificity can be extended to include other phylogenetic and geographic dependencies [42]. In any case, host specificity should be adapted to the potential host trees and/or substrates present in the specific system and geographical region studied. For example, aspen *Populus tremula* may function as a surrogate tree for cothreatened *F. excelsior* affiliate lichens in other Swedish and European geographical regions [43]. Our projections showed that affiliated generalist species with low tree host specificity had lower average coextinction probabilities (*Ā*) and were subsequently more resistant to epidemic tree death in mixed wooded stands. This complies with the diversity resistance hypothesis, which argues that diverse communities are more resilient to disease and pests (e.g., [44]). As such, diverse host tree assemblages and affiliate communities clearly represent an important “line of defense” and route to quicker recovery when faced with EIDs.

The impacts of tree deaths and host specificity are not always cleanly segregated from other drivers of affiliate extinction. For example, suitable semi-open and humid deciduous lichen habitats have been declining in large parts of Europe due to intensive forestry and reduced forest grazing. Disentangling coextinction effects from other causes of extinction and community change, such as habitat loss and climate change, has important conservation implications and should be the focus of future research (Fig. 1) [19]. For this study, we have assumed the simplest scenario, with ash dieback tree death being the single casual factor in affiliate extinctions, recognizing that host specificity may vary considerable throughout the distribution range of the affiliate and interact with co-occurring threats to *F. excelsior* and alternative host trees. In Europe there is a number of co-occurring tree pathogens that could compound the ecological impacts of ash dieback disease in deciduous wooded habitats [23]. The Dutch elm disease (DED) is probably the most prominent and well-known example and was first detected on Gotland in 2005. Despite efforts to eradicate DED on Gotland, both small-leaved elm *Ulmus minor* and *F. excelsior* became red-listed in Sweden in 2010 [21]. Both tree species represent key habitats for epiphytic biodiversity and share many red-listed lichens [23], whereby their compounding effects on affiliate coextinctions may be severe. Forest conservationists should account for the interplay of co-occurring tree diseases as well as the loss of host trees through inappropriate management and habitat destruction [2]. Tree epidemics are clearly not unique to Europe, but threaten trees and affiliate biodiversity on a global basis. Chestnut blight, caused by the pathogen *Cryphonectria parasitica*, was introduced in the US from Asia in the late 19^th^ century. The blight spread rapidly across the range of chestnut, and within 50 years had converted this foundation tree to a rarely flowering understory shrub across approximately 3.6 million ha (e.g., [14]). The impact of Chestnut blight on affiliate biodiversity and ecosystem function was never documented. Pine wilt disease, caused by the North American pine wood nematode, *Bursaphelenchus xylophilus*, is an example of a serious emerging disease in both Asian and European forests [45]. In view of the global biodiversity crisis, where at least 10% of the world’s trees are threatened with extinction [46], it is imperative that coextinction threats from epidemic tree death are accounted for.

Our example focus on ash dieback disease and lichens, but the general patterns are broadly representative of diseased host tree populations and their affiliates in forests throughout the world. Rapid responses in terms of anticipatory scenario planning and risk analysis based on local host and affiliate matrix data are useful tools to project future coextinction threats in various forested environments. Coextinction models dealing with EIDs should take into account that (i) affiliates do not occur uniformly on trees in relation to variables such as tree age, size, microclimate and locality; (ii) affiliates can go extinct from host tree declines; (iii) affiliates with intermediate host specificity can be cothreatened; (iv) coextinction threats varies locally and regionally, interacting with other co-occuring threats; and (v) affiliate individuals co-occurring on other tree species may be genetically distinct varieties or subspecies (Fig. 1).Results can form a basis for remedial management and national strategies which contend with the wider implications of EIDs on trees, especially in relation to valuable natural and cultural habitats, which are still lacking and urgently needed.

## Supporting Information

Figure S1 NMDS plot (2D, stress: 0.08) of site-level species composition on *Fraxinus excelsior* of pre epidemic lichen communities (filled circles) and average composition values projected under the most likely ash dieback outbreak scenario (open circles). Stands not substantially different from pre-epidemic communities in the ANOSIM (Table 1) are displayed by their site names.
(TIF)

Appendix S1 The most optimistic and likely scenario projections of average coextinction probabilities (*Ā*) of the 174 epiphytic lichen study species on *Fraxinus excelsior* populations and mixed tree species populations, respectively, where n  =  number of stands where the species was recorded. Host specificity (*s*) is the fraction of lichen records occurring on *F. excelsior* in relation to the total number of observations on all host trees on Gotland. Species are arranged in descending order of their average coextinction probabilities (*Ā*) at the most likely *F. excelsior* mortality perturbations among all tree species. Red-listed species are marked in bold. Species with asexual dispersal via symbiotic propagules such as soredia, blastidia or isidia, or via thallus fragmentation, are marked with three asterisks (***).
(XLSX)
