# Supplementary figures and images for: Estimating Coextinction Risks from Epidemic Tree Death: Affiliate Lichen Communities among Diseased Host Tree Populations of Fraxinus excelsior
Source: PLoS One. 2012 Sep 25;7(9):e45701. doi: 10.1371/journal.pone.0045701 (PMC3458109; doi:10.1371/journal.pone.0045701)

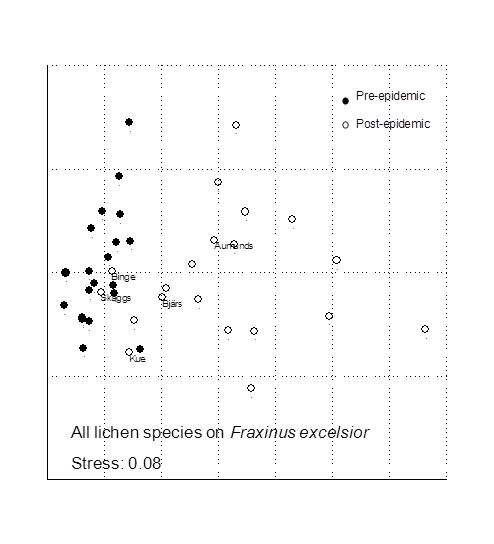

Supplement: Figure S1 — NMDS plot (2D, stress: 0.08) of site-level species composition on Fraxinus excelsior of pre epidemic lichen communities (filled circles) and average composition values projected under the most likely ash dieback outbreak scenario (open circles). Stands not substantially different from pre-epidemic communities in the ANOSIM (Table 1) are displayed by their site names. (TIF) [file pone.0045701.s001.tif]
